# Supplementary material for: MetaRibo-Seq measures translation in microbiomes
Source: Nat Commun. 2020 Jun 29;11:3268. doi: 10.1038/s41467-020-17081-z (PMC7324362; doi:10.1038/s41467-020-17081-z)
Supplement: Supplementary file 10 — Supplementary Data 7 [file 41467_2020_17081_MOESM10_ESM.zip › File2/Confidence_High_Taxonomy/108053_out.krona.html]

Javascript must be enabled to view this page.

members
magnitude
magnitudeUnassigned
count
unassigned
taxon
rank

108053\_out

40

2
40
superkingdom

40
phylum
976

200643
class
40

171549
order
40

171552
family
40

838
40
genus

3
species
2292365

SRS012849\_contig\_number\_1805SRS015794\_contig\_number\_3243SRS047044\_contig\_number\_7435

species
26

SRS011134\_contig\_number\_31209SRS012969\_contig\_number\_contig-100\_355.155143SRS019285\_contig\_number\_420SRS044535\_contig\_number\_19252SRS049712\_contig\_number\_20140SRS049959\_contig\_number\_359SRS049959\_contig\_number\_26271SRS049995\_contig\_number\_34217SRS065397\_contig\_number\_13522SRS074586\_contig\_number\_4994SRS078176\_contig\_number\_18954SRS078419\_contig\_number\_28560SRS097958\_contig\_number\_5973SRS104036\_contig\_number\_233SRS1041031\_contig\_number\_26335SRS1041134\_contig\_number\_9178SRS1041137\_contig\_number\_contig-100\_220.175641SRS1041140\_contig\_number\_22453SRS104327\_contig\_number\_contig-100\_3082.3083SRS104912\_contig\_number\_20423SRS105082\_contig\_number\_1698SRS143876\_contig\_number\_24010SRS144297\_contig\_number\_2281SRS144537\_contig\_number\_35872SRS144714\_contig\_number\_15145SRS148253\_contig\_number\_1853
165179

species
3
1263102

SRS016541\_contig\_number\_17935SRS017307\_contig\_number\_22743SRS054059\_contig\_number\_2213


SRS012969\_contig\_number\_11447SRS023526\_contig\_number\_375SRS077641\_contig\_number\_contig-100\_1233.122464SRS077849\_contig\_number\_23264
2292054
species
4

species
4
59823

SRS013521\_contig\_number\_938SRS013687\_contig\_number\_30889SRS077641\_contig\_number\_18127SRS143181\_contig\_number\_6723
